# Development and reliability of a systematic method to evaluate lumbar paraspinal muscle size and quality in computed tomography images

**DOI:** 10.1016/j.xnsj.2025.100822

**Published:** 2025-11-19

**Authors:** Cristiane R. Carlesso, Jaclyn M. Sions, William J. Anderst, Charity G. Patterson, Rafaela M. Barbosa, Luiza P. Weissmann, Tom Gale, Michael J. Schneider, Sara R. Piva

**Affiliations:** a Department of Physical Therapy & Athletic Training, University of Utah, 383 Colorow Dr., Salt Lake City, UT 84108, United States; b Department of Physical Therapy, University of Delaware, STAR Health Sciences Complex, 540 S College Ave, Newark, DE 19713, United States; c Department of Orthopedic Surgery, University of Pittsburgh, 3820 South Water Street, Pittsburgh, PA 15206, United States; d Department of Physical Therapy, University of Pittsburgh, Bridgeside Point 1, 100 Technology Drive, Pittsburgh, PA 15219, United States; e Department of Community Health Services & Rehabilitation Sciences, University of Pittsburgh, Bridgeside Point 1, 100 Technology Drive, Pittsburgh, PA 15219, United States

**Keywords:** Paraspinal muscles, Computed tomography, Low back pain, Reproducibility of results, Reliability, Muscle imaging analysis

## Abstract

**Background:**

Lumbar paraspinal muscles (LPMs) are critical for spinal stability, and their morphological alterations are associated with chronic low back pain (cLBP). Computed tomography (CT) offers a feasible alternative to magnetic resonance imaging for assessing LPM morphology; however, its broader use has been hindered by inconsistent measurement methods and limited reliability data. This study aimed to describe a systematic CT-based method for evaluating LPM cross-sectional area (CSA) and attenuation (MA) in adults with cLBP and to determine its intra and inter-examiner procedural reliability.

**Methods:**

High-resolution CT images were used to analyze bilateral LPMs: erector spinae (L1–L5), multifidi (L1–S1), psoas (L3), and quadratus lumborum (L3). Two trained examiners independently performed image selection and analysis, blinded to subject identity and prior results. Procedural reliability was quantified using intraclass correlation coefficients (ICC_2,1_) with 95% confidence intervals (CI). Mean ICCs<0.50 indicated poor, 0.50–0.74 moderate, 0.75–0.90 good, and >0.90 excellent procedural reliability.

**Results:**

Thirty-six participants (40% female; age 48±15 years; BMI 27.2±4.3 kg/m²) with moderate disability were included. Intraexaminer procedural reliability was excellent for CSA of the erector spinae, psoas, and quadratus lumborum (ICC point estimates >0.916, >0.959, and >0.962, respectively); and good to excellent for multifidi CSA (ICC point estimates: 0.770–0.916), with greater variability at L1 and L2. Inter-examiner procedural reliability was excellent for CSA of the erector spinae, psoas, and quadratum lumborum (ICC point estimates >0.916, >0.946, and >0.941, respectively); and moderate to excellent for multifidi CSA (ICC point estimates: 0.593–0.945), with lower reliability at upper lumbar levels. Intra and inter-examiner procedural reliability for MA was excellent across all LPMs.

**Conclusions:**

The presented CT-based protocol provides a standardized and reproducible method for assessing LPM CSA and MA in adults with cLBP. Demonstrated high procedural reliability—including image selection and analysis—supports its use for consistent evaluation of LPM morphology in both clinical and research contexts, particularly where muscle degeneration complicates image-based assessment.

## Background

Chronic low back pain (cLBP) is a leading cause of disability and reduced physical function worldwide [1,2]. Evidence suggests that lumbar paraspinal muscles (LPMs) play a role in the development and persistence of cLBP [3,4]. Compared to healthy controls, individuals with cLBP often present with smaller and fatter LPMs [[5], [6], [7]], underscoring the importance of reliably assessing these muscles in clinical research to understand their impact in cLBP.

Computed tomography (CT) and magnetic resonance imaging (MRI) are considered the gold standards for evaluating muscle morphology [8,9]. Both imaging modalities allow assessment of LPM size, measured by cross-sectional area (CSA), and muscle quality, estimated by mean voxel attenuation in Hounsfield units (HU) on CT or proton density fat fraction on MRI. In healthy individuals LPM size and quality were highly correlated between these imaging modalities (Pearson *r*>0.90) [8]. CT, however, offers greater accessibility, lower cost, and fewer contraindications—particularly for individuals with metal implants—despite involving low radiation exposure [[10], [11], [12]]. Moreover, CT imaging provides validated reference values for muscle quality, making it a practical tool for studying LPM degeneration.

Despite its advantages, CT use for evaluating LPM morphology in cLBP remains limited. Most existing studies focus on measurement reliability—muscle analysis using preselected images—rather than procedural reliability, which includes the entire imaging workflow of acquisition, image selection, and muscle analysis [[13], [14], [15]]. Procedural reliability is potentially less frequently assessed due to the additional time and effort required. However, procedural reliability provides a more accurate reflection of real-world clinical and research conditions, where examiner decisions during the image selection can influence muscle measures.

Methodological inconsistencies further limit comparability across studies [16]. For example, many studies focus on a single spinal segment, which may not accurately represent overall LPM degeneration [17]. Few studies specify whether imaging slices are perpendicular to the muscle, a critical factor for accurately measuring muscle CSA. Additionally, many studies assess only small portions of the muscle for attenuation, neglecting the uneven distribution of fat within the LPMs [18]. Finally, while the LPMs have distinct functional roles (ie, extension versus rotation), several studies treat the LPMs as a single unit rather than assessing muscles individually [19,20]. And, to our knowledge, no literature substantiates standard CT imaging approaches for assessing LPMs individually, in contrast to the methodological guidelines available for MR imaging [[21], [22], [23]].

To address these gaps, this research aims to describe a systematic CT-based method for quantifying LPM CSA and attenuation in adults with cLBP and to evaluate its intra and inter-examiner procedural reliability. Establishing a standardized and reliable CT analysis protocol may improve the consistency and interpretability of research examining paraspinal muscle degeneration in adults with cLBP.

## Methods

This study utilized CT images from a subset of participants with cLBP enrolled in a prospective observational cohort study at the University of Pittsburgh [24]. The parent study was approved by the Institutional Review Board, which also covered this secondary analysis, and all participants provided informed consent. CT scans were acquired between January 2021 and March 2024 at the University of Pittsburgh Medical Center (UPMC) Mercy Hospital. This study follows the Guidelines for Reporting Reliability and Agreement Studies (GRRAS) [25].

### Participants

Inclusion criteria for the parent study were ≥18 years old, back pain located between the inferior border of the ribcage and gluteal fold for more than 3 months, occurring on at least half the days in the past 6 months [26], ability to speak and understand English, and willingness to comply with all study procedures. Exclusion criteria included the inability to identify the participant in the affiliated UPMC health record system, participation in a masked interventional study for low back pain, and medical conditions that would either preclude safe participation or result in noncompliance. Specific exclusions for CT imaging acquisition were pregnancy and multilevel lumbar spinal fusion, as well as 2 criteria specific to the parent study, ie, body mass index (BMI) ≥35 kg/m² (due to the difficulty of imaging during dynamic movements), and inability to perform lumbar movements required for spinal segmental motion analysis [27].

### Computed Tomography Imaging

Participants underwent high-resolution CT imaging of the lumbar spine (L1-S1), with an average voxel size of 0.29×0.29×0.625 mm and a slice thickness of 0.625 mm, using a peak voltage of 120 kVp. Imaging was conducted with individuals in a supine position using a GE Discovery CT750 HD CT scanner (Chicago, IL). The average radiation exposure was estimated at 21 mSv, which falls within the safe annual radiation dose range [11]. This estimate was calculated using DLP (mGy-cm) from the scanner and a conversion k-factor [28]. A bone density phantom (Mindways Inc., Austin, TX) was used to account for variations in radiation exposure due to differences in body size and changes in tissue density across the lumbar spine. Imaging was performed by trained staff following standardized procedures. CT images stored in Digital Imaging and Communications in Medicine (DICOM) format were imported into Mimics software (Materialise, version 26) and processed on a high-performance desktop computer (Dell Precision 3650 Tower with dual 68.6 cm monitors) in a quiet, unlit room.

CT images were considered for LPM assessment if they were free from metal artifacts and provided sufficient image quality to allow for full visualization of the muscles. The muscles targeted for measurement included the bilateral multifidi, erector spinae, quadratus lumborum, and psoas muscles. Following previous studies [17,29], the multifidi and erector spinae muscles were measured bilaterally from L1 to L5 levels. For the multifidi, we also included the S1 level, as these muscle fascicles attach to the sacrum and play a role in stabilizing and controlling L5-S1 segmental motion. The quadratus lumborum and psoas muscles were measured only at the L3 level due to the difficulty in identifying the quadratus lumborum muscle at higher levels and the relatively consistent function of the quadratus across all levels of the lumbar spine [13,30,31].

### Image Reslicing Process

**Fig. 1:**
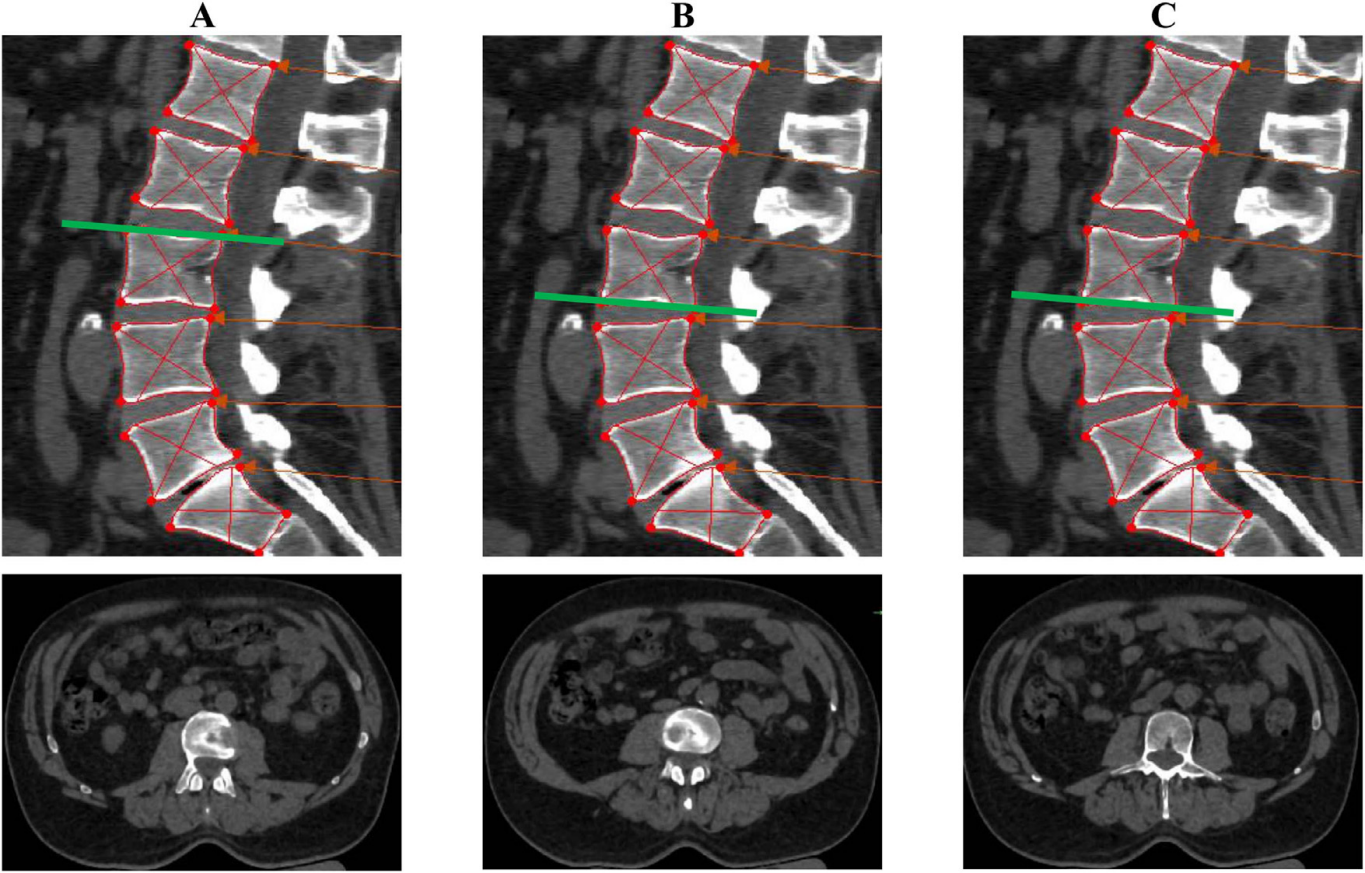
Tested reslicing methodologies. Examples of alternative reslicing approaches before muscle measurement. Top: sagittal views; bottom: corresponding axial slices. Red lines outline vertebrae to locate centroids (the intersection of the lines); green lines mark reslice positions. Reslicing at (A) upper or (B) lower vertebral endplates lacks clear axial landmarks. (C) Mid-vertebral body reslicing reveals spinous and transverse processes, providing bony landmarks for measurement.

The first methodological decision was to select the image slice that best captured bony landmarks in an anatomical axial view for each lumbar spinal segment, thereby optimizing LPM identification and ensuring perpendicular alignment of muscle fascicles for accurate CSA measurements. As there were no established guidelines for slice selection in CT imaging, we considered 3 reslicing methodologies to optimize landmark identification: at the (1) upper endplate, (2) lower endplate, and (3) mid-vertebral body (Fig. 1). These options were systematically evaluated from levels L1 to S1 using CT images from 10 individuals with cLBP.

To determine the optimal slices, we first outlined the vertebral bodies from L1 to S1 in the central sagittal view using a software drawing tool that automatically displays the vertebral centroid, ie, the point where the intersectional lines intersect. We then used the Interactive Multiplanar Reconstruction (IMPR) tool to draw lines parallel to the upper and lower endplates, as well as the mid-vertebral body at the vertebral centroid. The exact angle of the IMPR line for the upper endplate was used for the mid-vertebral body, as the upper endplate generally has fewer degenerative irregularities than the lower endplate [32].

**Fig. 2:**
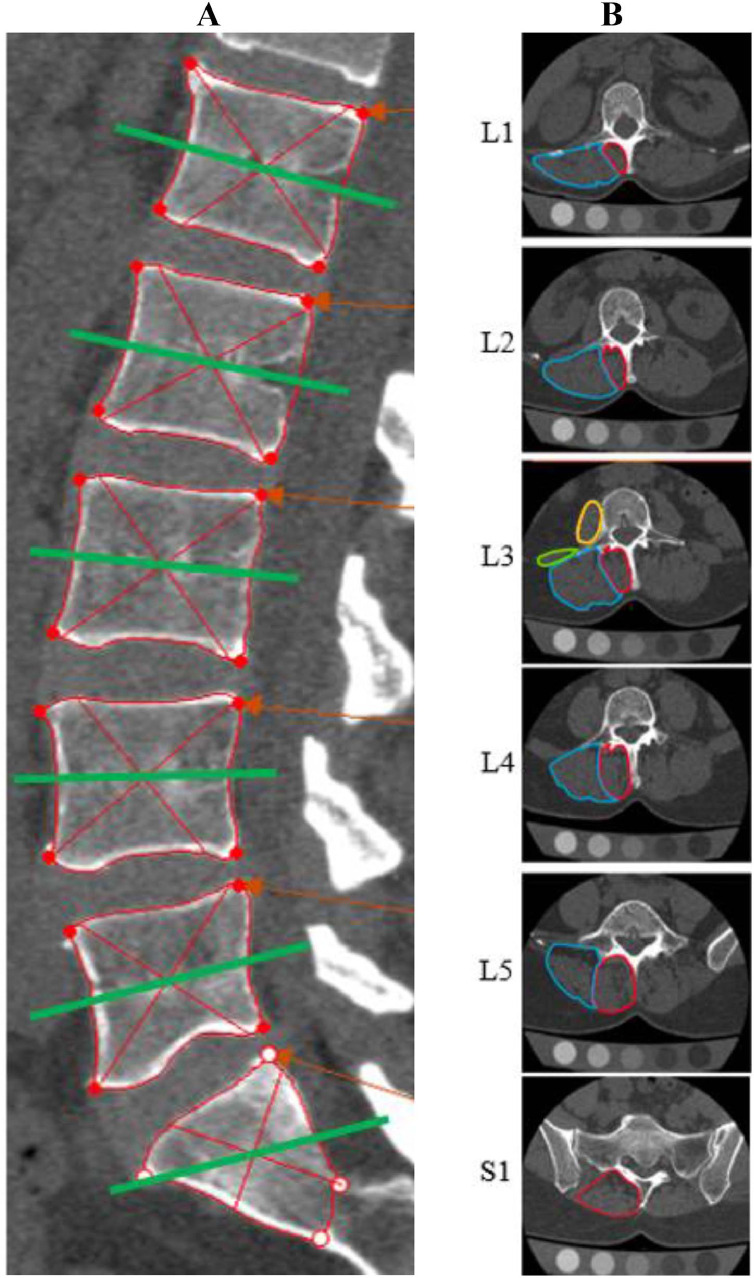
Selected imaging reslicing method over the mid-vertebral body. (A) Sagittal view showing the reslicing process. Red lines outline vertebrae to locate centroids (intersection points), with green lines aligned to the superior endplate at each centroid. For the sacrum, the line from the vertebra above is replicated. (B) Axial view of corresponding resliced segments, with unilateral muscle representations: multifidus (red), erector spinae (blue), quadratus lumborum (green), and psoas (yellow).

After analyzing the results of the reslicing testing, we discarded the upper endplate slice because it did not capture the transverse processes (Fig. 1, panel A). We also discarded the lower endplate method, which frequently missed bony landmarks and produced inconsistent perpendicular lines to the LPMs due to degenerative changes (Fig. 1, panel B). The mid-vertebral body slice was the most effective method because it frequently included the spinous and transverse processes, facilitating the identification of muscle borders (Fig. 1, panel C). This reslicing process was consistent from L1 to L5 levels. At the S1 level, given the variability in sacral slope angles among individuals, we duplicated the L5 IMPR line and adjusted it to the S1 centroid to prevent overlap over the paravertebral muscles between the S1 and L5 slices (Appendix 1). The reslicing process performed in the sagittal view and the obtained slices across the lumbar area in the axial view are illustrated in Fig. 2, panels A and B.

### Image Analysis Process

**Fig. 3:**
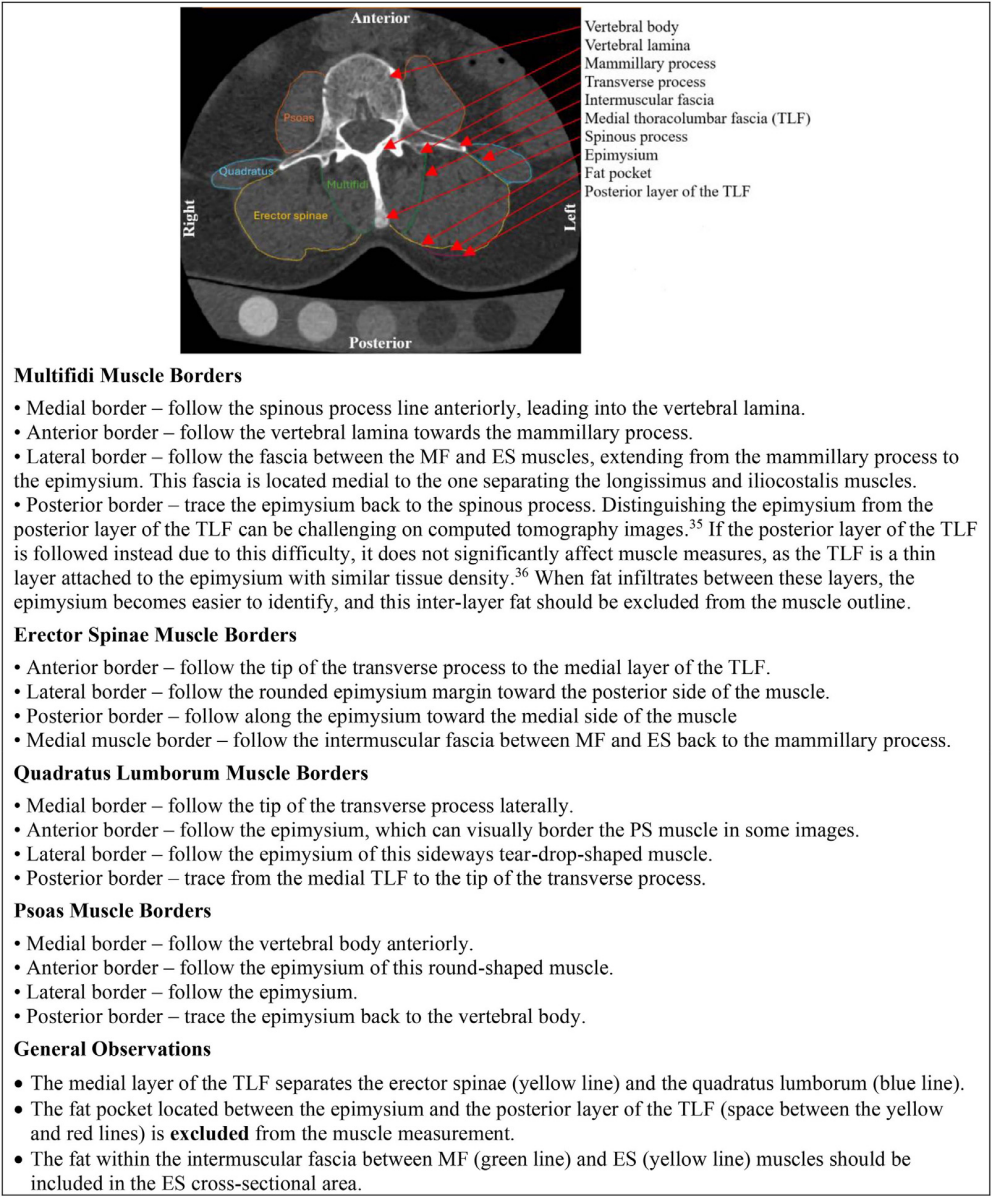
General guidance to outline the lumbar paraspinal muscles based on anatomical landmarks. Anatomical landmarks used to identify borders of each lumbar paraspinal muscle. ES, erector spinae; MF, multifidi ; PS, psoas ; TLF, thoracolumbar fascia [34,35].

**Fig. 4:**
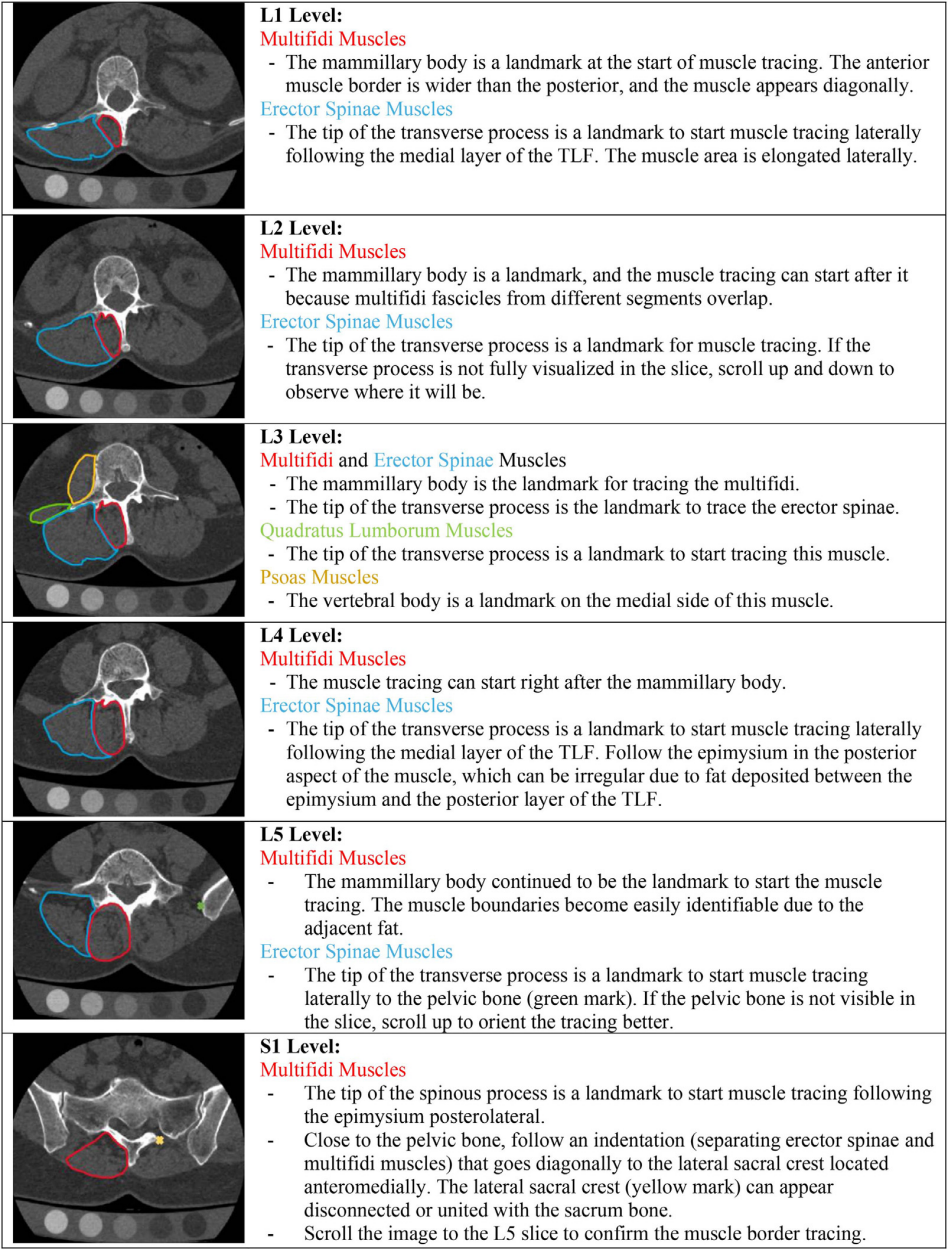
Specific guidance to outline the lumbar paraspinal muscles at each lumbar spinal segment. Illustration showing paraspinal muscle borders at each lumbar level. Multifidus cross-sectional area increases from the upper to lower lumbar segments, while erector spinae cross-sectional area decreases. TLF, thoracolumbar fascia.

**Fig. 5:**
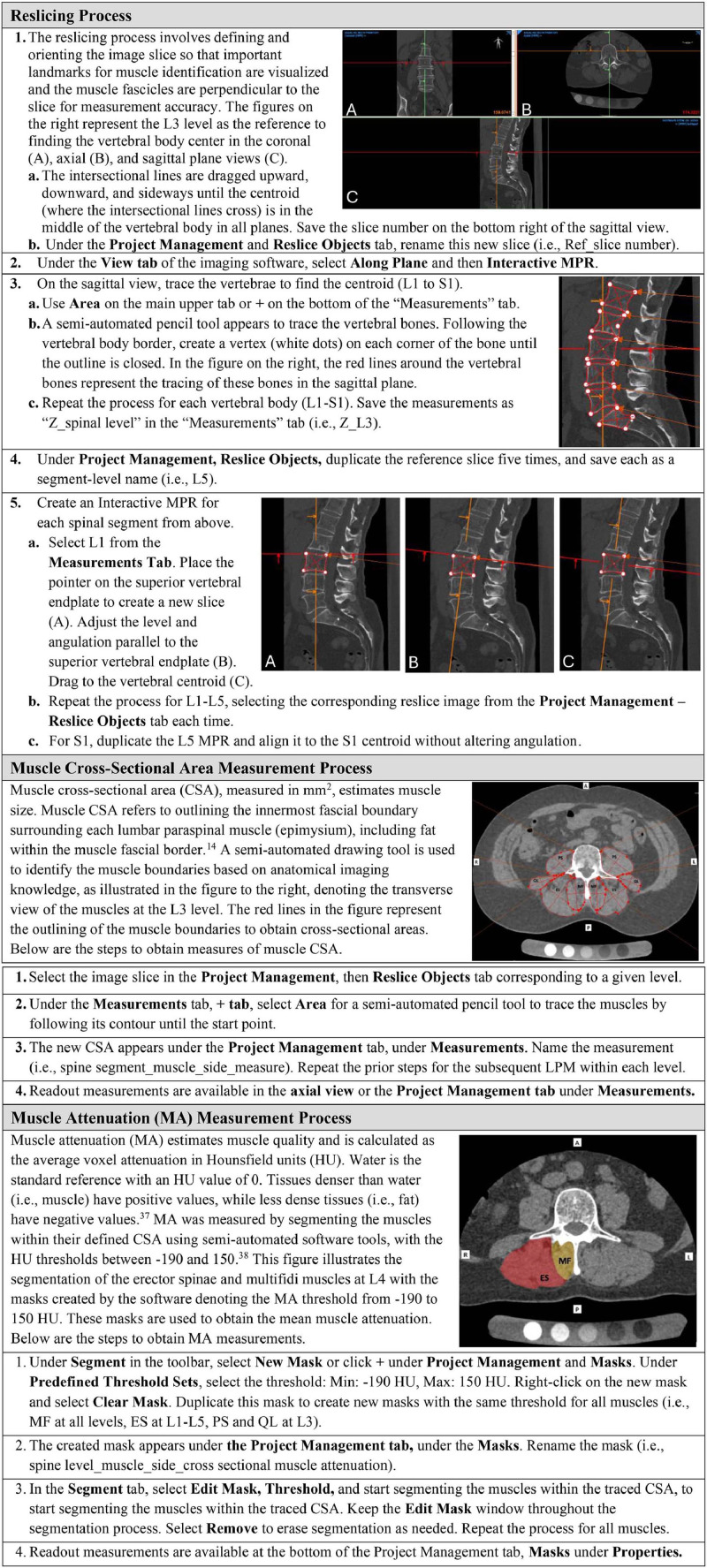
Systematic method for analyzing lumbar paraspinal muscles using computed tomography images. This workflow, implemented with Mimics software, illustrates the assessment of muscle cross-sectional area and attenuation as presented in the study. A, anterior; P, posterior; L, left; R, right; MF, multifidi; ES, erector spinae; QL, quadratus lumborum; PS, psoas [13,36,37].

Muscle borders in individuals with low back pain are more irregular compared to those in healthy individuals, due to muscle atrophy and fat infiltration [33]. To address this, we reviewed the literature on imaging modalities standards, consulted online 3-dimensional anatomy atlases (ie, Elsevier Complete Anatomy App), and performed muscle dissections in cadaver specimens to develop guidelines for outlining the LPMs in individuals with cLBP (Fig. 3). Additionally, we considered the unique anatomical variations of each lumbar segment in the axial plane [34,35], and reported these distinctive features to ensure consistent identification of LPMs across the spinal levels (Fig. 4). Given the absence of detailed step-by-step procedures for CT image analysis using available software, we systematically describe how to utilize the imaging software tools (ie, Mimics used as the reference) to measure LPM CSA and MA (Fig. 5), following established CT imaging parameters [36,37]. After obtaining muscle measurements, values were manually imported into a designated project using REDCap data management software.

### Reliability Testing Protocol

Inter- and intra-examiner procedural reliability for measuring LPM CSA and MA in individuals with cLBP was evaluated using the CT imaging analysis method described in this study. Two examiners, with 5 and 12 years of clinical experience in musculoskeletal physical therapy, independently assessed the muscles bilaterally. Examiners were blinded to each other's results, prior measurements, and subject identifiers. Before the study, the examiners underwent approximately 20 hours of training in CT imaging analysis using Mimics software.

To ensure examiner independence, a separate researcher, not involved in the LPM assessments, assigned unidentified CT images from adults with cLBP sampled with a balanced distribution of age and sex, to the examiners. To prevent prior image exposure and assessment bias, images used to develop the analysis method were excluded from reliability testing. Examiners were not allowed to revisit images once measurements were processed. To minimize anchoring bias—where the memory of the first assessment may influence the judgment of the second—the second muscle analysis for intraexaminer reliability testing was conducted at least 1 month, but no more than 2 months, after the first. Examiners followed standardized procedures for selecting image slices from which muscle measurements were taken. This process involved defining the image slices that captured the most perpendicular cross-section of the muscles for evaluation.

### Power Estimates

With 2 observations per participant, a sample size of 36 participants provides 80% power to detect an intraclass correlation (ICC) of 0.750 for assessing intra and inter-examiner procedural reliability. The power analysis was conducted under the alternative hypothesis (ICC=0.750) compared to the null hypothesis (ICC=0.500), using an F-test with a significance level of 0.05and conducted in NCSS/PASS version 15.0.13 [38].

### Statistical Analysis

Descriptive statistics were used to characterize the study sample. A 2-way random effects model for absolute agreement between single measurements—Intraclass Correlation Coefficient (ICC_2,1_) with a 95% confidence interval (CI)—was used to assess inter- and intraexaminer procedural reliability. This model was selected because each muscle measurement was independently obtained by each examiner (subscript number 2), the aim was to assess agreement on single measurements (subscript number 1), and examiners were treated as random samples from broader populations able to perform these muscle assessments [39]. ICC_2,1_ values <0.50 indicate poor reliability, 0.50–0.74 moderate reliability, 0.75–0.90 good reliability, and >0.90 excellent reliability. Standard errors of measurements were calculated for pooled data to indicate measurement variability, which can inform clinical applications [40]. Calculations were performed as ((SEM=SD*√(1-r)), where SD represents the standard deviation of the repeated measures scores and r represents the reliability of the measurement, in this case, the ICC point estimate. Analyses were conducted using SPSS software (IBM Corp., Chicago, IL, Version 26.0).

## Results

Of the 80 participants with available CT images in the parent study, 10 were used solely for the development of the imaging analysis method. Five were excluded due to metal artifacts, 4 because the targeted muscles were not fully captured, and 2 due to poor image quality. From the remaining 59 participants with acceptable images, 36 were selected for reliability testing with a relatively balanced distribution of age and sex. The remaining 23 were not assessed once the required sample size was reached based on power estimates. (Appendix 2). Participants in this study had an average age of 48.6±14.7 years, a BMI of 27.2±4.2 kg/m², and moderate LBP-related disability (Oswestry Disability Index: 22±11) [41]. Forty percent were female, and 75.0% were White (Table 1).

**Table 1:** Demographic and clinical characteristics of the study sample.

Individual characteristics	*N*=36
Age (years), mean±SD	48.6±14.7
Height (m), mean±SD	1.7±0.1
Weight (Kg), mean±SD	79.9±15.1
Body mass index (kg/m^2^), mean±SD	27.2±4.2
Sex at birth, N (%)	
Female	16 (44)
Male	20 (56)
Race, N (%)	
White	27 (75.0)
African American	7 (19.4)
Asian	1 (2.8)
Not reported	1 (2.8)
Education, N (%)	
Did not complete High School	17 (47.2)
High School Degree	4 (11.1)
College or Baccalaureate Degree	13 (36.1)
Associate’s, Technical, or Post-Graduate Degree	2 (5.6)
How long is back pain ongoing, N (%)	
>3 but <6 months	1 (2.8)
6 months to <1 year	5 (13.9)
1 to <5 years	10 (27.8)
>5 years	20 (55.6)
Oswestry Disability Index (0 to 100), mean±SD	22.1±10.6

Intraexaminer procedural reliability for the erector spinae CSA and MA measurements demonstrated excellent reliability (ICC point estimates>0.916) across lumbar levels (Table 2). The psoas measurements at L3 also showed excellent intraexaminer reliability (ICC point estimates>0.959). The quadratus lumborum CSA measurements at L3 indicated excellent intraexaminer reliability (ICC point estimate>0.962), while the MA measurements on the left side were lower, achieving only good reliability (ICC=0.883; 95%CI: 0.783, 0.938). Reliability for the multifidi muscles was more variable. Excellent intraexaminer reliability was found for multifidi MA measurements across all spinal levels (ICC point estimates=0.914–0.995) and CSA measurements at L5 on the right side (ICC=0.916; 95%CI: 0.827, 0.958). Good intraexaminer reliability was observed for right and left multifidi CSA measurements at all other levels and L5 on the left side (ICC point estimates=0.768–0.881).

**Table 2:** Intraexaminer procedural reliability for lumbar paraspinal muscle measurements.

(*N*=36)			Left side				Right side			
			Mean±SD		Reliability		Mean±SD		Reliability	
			Assessment 1	Assessment 2	ICC (95% CI)	SEM	Assessment 1	Assessment 2	ICC (95% CI)	SEM
CSA(mm^2^)	ES	L1	2138±515	2114±533	0.959 (0.922, 0.979)	106.10	2113±573	2078±564	0.945 (0.895, 0.971)	133.33
		L2	2202±564	2230±565	0.972 (0.946, 0.985)	94.46	2187±566	2208±559	0.969 (0.940, 0.984)	99.04
		L3	2070±530	2065±525	0.982 (0.965, 0.991)	70.77	2023±532	2043±514	0.983 (0.968, 0.991)	68.19
		L4	1732±458	1684±438	0.919 (0.846, 0.958)	127.50	1648±452	1654±448	0.956 (0.916, 0.977)	94.39
		L5	1204±338	1230±334	0.916 (0.843, 0.956)	97.38	1185±319	1217±294	0.949 (0.899, 0.974)	69.22
	MF	L1	419±105	399±103	0.828 (0.683, 0.910)	43.13	408±98	394±105	0.873 (0.764, 0.933)	36.17
		L2	588±121	553±121	0.775 (0.564, 0.885)	57.40	582±121	545±121	0.770 (0.549, 0.883)	58.03
		L3	815±160	809±183	0.855 (0.735, 0.924)	65.31	821±179	808±198	0.881 (0.780, 0.937)	65.03
		L4	1127±210	1130±290	0.793 (0.630, 0.889)	113.74	1146±239	1131±270	0.837 (0.705, 0.914)	102.75
		L5	1330±286	1272±253	0.878 (0.741, 0.940)	94.13	1325±277	1280±289	0.916 (0.827, 0.958)	82.02
		S1	1012±183	950±183	0.768 (0.519, 0.886)	88.14	995±215	940±191	0.871 (0.679, 0.942)	72.91
	PS	L3	1140±346	1157±356	0.959 (0.921, 0.979)	71.07	1056±274	1052±275	0.981 (0.963, 0.990)	37.84
	QL	L3	549±219	545±220	0.977 (0.955, 0.988)	33.29	497±172	474±164	0.962 (0.900, 0.983)	32.75
MA(HU)	ES	L1	40.38±11.18	40.49±11.29	0.992 (0.983, 0.996)	1.00	40.52±13.06	40.51±13.15	0.995 (0.990, 0.997)	0.93
		L2	38.93±13.32	38.39±13.56	0.993 (0.985, 0.996)	1.12	38.59±13.13	38.95±13.10	0.993 (0.985, 0.996)	1.10
		L3	36.32±13.93	36.39±14.05	0.996 (0.991, 0.998)	0.88	36.54±13.27	36.55±13.54	0.988 (0.976, 0.994)	1.47
		L4	33.75±16.25	34.22±15.45	0.980 (0.961, 0.990)	2.24	31.80±15.41	32.08±16.01	0.988 (0.977, 0.994)	1.72
		L5	14.90±20.51	14.25±20.92	0.983 (0.967, 0.991)	2.70	11.53±21.48	11.42±22.25	0.970 (0.941, 0.984)	3.79
	MF	L1	37.48±15.39	35.55±7.20	0.959 (0.921, 0.979)	2.29	36.16±17.03	35.55±17.56	0.971 (0.944, 0.985)	2.95
		L2	35.32±16.88	35.28±18.08	0.955 (0.914, 0.977)	3.71	35.74±14.46	35.67±16.67	0.963 (0.930, 0.981)	2.99
		L3	38.57±17.47	39.30±18.48	0.982 (0.965, 0.991)	2.41	36.81±17.19	37.48±17.47	0.977 (0.956, 0.988)	2.63
		L4	35.91±19.68	36.12±21.19	0.977 (0.956, 0.988)	3.10	35.00±18.40	35.39±18.55	0.977 (0.955, 0.988)	2.80
		L5	30.10±21.99	30.52±22.35	0.990 (0.980, 0.995)	2.22	28.43±19.83	28.95±20.15	0.985 (0.972, 0.992)	2.45
		S1	27.55±24.26	29.20±24.77	0.986 (0.969, 0.993)	2.90	25.39±23.95	26.81±25.27	0.980 (0.961, 0.990)	3.48
	PS	L3	40.24±12.68	40.44±12.66	0.977 (0.955, 0.988)	1.92	43.12±11.02	43.84±11.15	0.982 (0.963, 0.991)	1.49
	QL	L3	40.38±8.63	40.92±8.00	0.883 (0.783, 0.938)	2.84	39.43±10.10	40.03±9.77	0.936 (0.878, 0.967)	2.51

CSA, cross-sectional area; MA, muscle attenuation; HU, Hounsfield unit; SD, standard deviation; ICC, intraclass correlation coefficient; CI, confidence interval; SEM, standard error of measurement; ES, erector spinae; MF, multifidi; QL, quadratus lumborum; PS, psoas.

Inter-examiner procedural reliability for the erector spinae CSA and MA measurements demonstrated excellent reliability (ICC point estimates>0.916) across all spinal levels (Table 3), as did the psoas CSA and MA measurements at L3 (ICC point estimates>0.946). The quadratus lumborum CSA and MA measurements at L3 showed excellent reliability (ICC point estimates=0.908–.946). As with intraexaminer reliability, the inter-examiner reliability for the multifidi muscles was variable. Excellent reliability was observed for multifidi MA measurements across all lumbar spinal levels (ICC point estimates=0.937–0.981), while CSA measurements showed excellent reliability only at L5 (ICC point estimates>0.935) and good reliability from L1 to L4 and at S1 (ICC point estimates=0.593–0.890).

**Table 3:** Inter-examiner procedural reliability for lumbar paraspinal muscle measurements.

(*N*=36)			Left side				Right side			
			Mean±SD		Reliability		Mean±SD		Reliability	
			Examiner 1	Examiner 2	ICC (95% CI)	SEM	Examiner 1	Examiner 2	ICC (95% CI)	SEM
CSA(mm^2^)	ES	L1	2144±517	2136±521	0.980 (0.960, 0.989)	73.40	2093±548	2105±541	0.980 (0.960, 0.989)	77.00
		L2	2192±570	2207±566	0.984 (0.969, 0.992)	71.85	2179±558	2169±544	0.968 (0.939, 0.984)	98.57
		L3	2055±528	2056±525	0.980 (0.960, 0.990)	74.46	2000±529	2046±534	0.967 (0.934, 0.983)	96.55
		L4	1750±476	1703±452	0.943 (0.890, 0.971)	110.78	1655±440	1647±448	0.943 (0.891, 0.971)	106.00
		L5	1214±356	1206±347	0.933 (0.873, 0.965)	90.98	1200±308	1176±323	0.916 (0.843, 0.956)	91.44
	MF	L1	431±97	405±95	0.694 (0.470, 0.832)	53.10	429±97	399±92	0.683 (0.442, 0.828)	53.21
		L2	601±126	587±118	0.593 (0.333, 0.769)	77.83	601±127	581±130	0.659 (0.428, 0.809)	75.04
		L3	831±162	816±173	0.793 (0.633, 0.889)	76.21	853±174	797±195	0.779 (0.558, 0.889)	86.73
		L4	1130±217	1123±249	0.882 (0.782, 0.938)	80.04	1146±247	1149±282	0.870 (0.760, 0.932)	95.37
		L5	1318±270	1319±286	0.945 (0.896, 0.972)	65.20	1325±279	1332±296	0.935 (0.876, 0.966)	73.30
		S1	1008±184	1018±193	0.870 (0.760, 0.931)	67.96	994±218	995±213	0.890 (0.796, 0.943)	71.47
	PS	L3	1150±350	1166±347	0.946 (0.898, 0.972)	80.98	1060±272	1064±289	0.975 (0.952, 0.987)	44.35
	QL	L3	551±221	561±234	0.946 (0.897, 0.972)	52.87	486±159	501±187	0.941 (0.887, 0.969)	42.02
MA(HU)	ES	L1	40.05±11.15	40.13±11.37	0.990 (0.981, 0.995)	1.13	40.51±13.10	40.24±13.20	0.989 (0.979, 0.994)	1.38
		L2	38.77±13.50	38.42±13.56	0.993 (0.987, 0.997)	1.13	38.54±13.43	38.82±12.37	0.984 (0.968, 0.992)	1.63
		L3	36.19±13.96	36.25±14.01	0.993 (0.987, 0.997)	1.17	36.30±13.54	36.76±12.92	0.986 (0.972, 0.993)	1.57
		L4	33.28±15.99	34.57±14.99	0.969 (0.938, 0.985)	2.73	31.37±15.66	32.04±14.83	0.983 (0.968, 0.992)	1.99
		L5	15.69±20.20	15.43±20.59	0.967 (0.937, 0.983)	3.70	11.21±21.43	13.11±21.15	0.954 (0.910, 0.976)	4.57
	MF	L1	37.52±15.21	36.60±17.03	0.943 (0.892, 0.970)	3.85	36.19±16.60	36.93±16.58	0.968 (0.938, 0.983)	2.97
		L2	35.82±16.91	35.36±17.57	0.937 (0.880, 0.967)	4.33	35.99±14.13	35.55±15.72	0.969 (0.941, 0.984)	2.63
		L3	39.07±17.14	38.49±19.46	0.965 (0.932, 0.982)	3.42	37.46±16.04	35.94±18.63	0.948 (0.899, 0.973)	3.95
		L4	35.95±20.55	35.25±19.72	0.981 (0.964, 0.990)	2.78	35.50±18.40	34.60±19.00	0.961 (0.926, 0.980)	3.69
		L5	30.70±22.20	29.86±21.13	0.970 (0.942, 0.984)	3.75	28.85±20.06	27.95±19.21	0.977 (0.955, 0.988)	2.98
		S1	28.11±23.68	26.72±24.83	0.978 (0.957, 0.989)	3.60	26.09±23.70	25.00±24.29	0.975 (0.951, 0.987)	3.79
	PS	L3	40.46±12.76	39.85±12.01	0.973 (0.949, 0.986)	2.04	43.27±10.83	42.64±11.26	0.975 (0.951, 0.987)	1.75
	QL	L3	40.20±9.28	39.77±9.16	0.913 (0.836, 0.954)	2.72	39.66±10.06	38.64±10.38	0.908 (0.827, 0.952)	3.10

CSA, cross-sectional area; MA, muscle attenuation; HU, Hounsfield unit; SD, standard deviation; ICC, intraclass correlation coefficient; CI, confidence interval; SEM, standard error of measurement; ES, erector spinae; MF, multifidi; QL, quadratus lumborum; PS, psoas.

The calculated SEMs for all muscle measurements within and between examiners generally accounted for approximately 10% of the mean measurements, indicating moderate variability when assessing the CSA and MA of the LPMs in adults with cLBP using the previously described CT image analysis method. An exception to this pattern was observed in the MA measurement of the erector spinae at L5 level, where SEM values ranged from ∼10% to 30% of the measurement mean. This higher relative error was primarily due to the greater variability (standard deviation) in these measurements, while the mean values remained consistent. As a result, the SEM expressed as a percentage of the actual measurement increased notably for MA at level L5.

## Discussion

This study developed CT imaging analysis methods by refining strategies to optimize the visualization of anatomical landmarks and improve the identification of muscle contours. The development process involved reviewing CT imaging studies and conducting interactive tests to determine the most effective method for visualizing and evaluating LPMs. To our knowledge, this is the first study to provide detailed, illustrated guidance for identifying and measuring muscle CSA and MA using CT imaging, applicable to Mimics and other imaging software. This guidance will help examiners distinguish muscle boundaries and reliably assess LPMs in individuals with cLBP.

Our study addressed a critical knowledge gap regarding the reliability of CT imaging for assessing LPM in individuals with cLBP. We provided evidence supporting the procedural reliability of the analysis method described in this study, which aims to better reflect how examiners perform muscle measurements in research and clinical settings. While procedural reliability typically introduces greater variability between measurements due to the need for defining imaging slices for each assessment, our study found excellent reliability per ICC point estimates in 79% (82/104), good reliability in 17% (18/104), and moderate reliability in only 4% (4/104) of the measurements. Notably, our intra and inter-examiner procedural reliability estimates are consistent with a previous study on measurement reliability in cLBP [15].

Several factors may have contributed to these favorable results despite the additional step of selecting the image slices fort muscle analises [15,42,43]. For instance, reslicing the images at the mid-vertebral level facilitated muscle identification, as this location allows for clear visualization of bony landmarks, such as the transverse and spinous processes, which aid in defining the muscle area. Additionally, the systematic and standardized muscle analysis method, supported by illustrated guidance, likely played a crucial role in achieving overall good to excellent reliability.

Specific factors may have contributed to the differences in tracing the multifidi muscles and consequent moderate inter-examiner reliability of the multifidi CSA measurements at the upper lumbar segments. The multifidi muscles are relatively small at these levels, meaning that even minor differences between repeated measurements can significantly impact reliability. For example, the area of the mammillary process, located on the anterolateral border of the multifidi muscle, as visualized in the axial view, can vary depending on the positioning or angulation of the image slice used for muscle measurements. If the slice captures a larger area of the mammillary process, the multifidi CSA appears smaller when compared to a slice with a smaller mammillary process. Additionally, the increased thickness of the supraspinous and interspinous ligaments at upper lumbar levels could have influenced the multifidi CSA measurements [44]. Multifidi muscles in the upper lumbar levels also exhibit higher attenuation than at the lower levels (ie, L5 and S1), making it challenging to distinguish muscles from adjacent ligaments, as these tissues have similar attenuation on CT images.

Despite moderate reliability for multifidi CSA at upper spinal levels, where multifidi muscles are smaller, all other reliability indices were consistently excellent across the lumbar spine. This strong reliability persisted even as the muscle CSA of the erector spinae decreased, and the multifidi CSA increased from the upper to the lower lumbar levels. Likewise, intra and inter-examiner procedural reliability remained excellent despite low erector spinae and multifidi quality at the lower lumbar levels, as indicated by lower muscle attenuation suggesting greater intramuscular fat infiltration. The reliability findings for the multifidi CSA, coupled with the observed anatomical and structural differences across the lumbar spine, support the need for assessing LPMs at all regional segments in future studies exploring the clinical implications of muscle morphology and their response to interventions for cLBP.

Our CT imaging analysis method for evaluating LPMs has several strengths. We refined existing methods to systematically obtain muscle measurements at each lumbar level and developed a reslicing process to ensure an axial view perpendicular to the muscle fascicles, which may more accurately measure muscle CSA. Additionally, we demonstrated good to excellent intra and inter-examiner procedural reliability for assessing LPMs by following a standardized, comprehensive analysis process, provided examiners have relevant anatomical knowledge and imaging training. This approach mirrors how muscle analysis is performed in clinical practice and research settings. Moreover, our study was conducted in adults with cLBP rather than healthy controls. LPM degeneration is typically more pronounced in patients with cLBP [[45], [46], [47]], which may pose measurement challenges not found in healthy controls. While muscle degeneration makes imaging analysis challenging due to irregular muscle borders and fat infiltration, reliably measuring LPM size and quality is critical, as these muscle aspects may influence physical function and contribute to disability in adults with cLBP.

This study also has limitations. First, we did not assess the reliability of measuring the quadratus lumborum and psoas muscles across the entire lumbar spine, but only at L3. For efficiency, we chose L3 because the aforementioned muscles are easily identifiable at this level. Additionally, multiple measurements of the quadratus lumborum and psoas muscles may not be necessary for imaging studies, as their function is not level-dependent, unlike the erector spinae or multifidi muscles. Another limitation is that our study involved 2 physical therapists with substantial anatomical knowledge performing the muscle measurements, and we cannot guarantee that the same intra and inter-examiner reliability would apply to examiners with other backgrounds. Third, our findings may not be applicable to adults with a BMI greater than 35 kg/m², as these individuals were excluded from the analyses. Finally, our procedural reliability did not include image acquisition in terms of participant scan and rescan, resulting in two unique sets of images. Analyzing two distinct sets of images may reduce procedural reliability and increase measurement error.

## Conclusion

This study presents a systematic CT image analysis method for evaluating LPM size and quality in adults with cLBP, supported by evidence of intra and inter-examiner procedural reliability. The method provides a standardized and reproducible approach for assessing LPMs, addressing challenges posed by degeneration and irregular muscle borders. This protocol offers a valuable tool for clinicians and researchers to reliably evaluate LPM morphology and establishes a foundation for using CT imaging to monitor muscle changes over time, enhancing our understanding of the role of LPMs in cLBP.

## Authorship Contribution

**Cristiane R. Carlesso:** conceptualization; data curation and analysis; funding acquisition, investigation; methodology; project administration; software; visualization; writing—original draft; reviewing/editing the manuscript. **Jaclyn M. Sion:** conceptualization; investigation; methodology; validation; visualization; reviewing and editing the manuscript. **William J. Anderst:** conceptualization; methodology; resources; software; validation; visualization; reviewing/editing the manuscript. **Charity G. Patterson:** analysis; methodology; validation; visualization; reviewing the manuscript. **Rafaela M. Barbosa**: investigation; validation; visualization; reviewing the manuscript. **Luiza P. Weissmann**: validation; visualization; reviewing/editing the manuscript. **Tom Gale**: visualization; reviewing/editing the manuscript. **Michael J. Schneider**: investigation; validation; visualization; reviewing/editing the manuscript. **Sara R. Piva**: conceptualization; investigation; methodology; project administration; resources; software; supervision; validation; visualization; reviewing/editing the manuscript.

## Declaration of Competing Interest

This research received the Ph.D. Student Award from the University of Pittsburgh School of Health and Rehabilitation Sciences in 2023.

## Appendix Supplementary materials

Image, application 1
