# Supplementary material for: Development and reliability of a systematic method to evaluate lumbar paraspinal muscle size and quality in computed tomography images
Source: N Am Spine Soc J. 2025 Nov 19;24:100822. doi: 10.1016/j.xnsj.2025.100822 (PMC12753261; doi:10.1016/j.xnsj.2025.100822)

Appendix 1. Assessment of interactive multiplanar reconstruction at S1

*The images illustrate tested alternatives for reslicing the scan at the S1 level. Red lines outline the vertebrae to identify their centroids.* ***(A)*** *Alternative 1: reslicing based on the upper endplate with the line placed the line at the mid-vertebra.* *This approach was problematic for high sacral slope angles, causing overlapping lines at the muscle level.* ***(B)*** *Alternative 2: reslicing by duplicating the L5 level line, resulting in slices more perpendicular to the muscles.*

| **A**  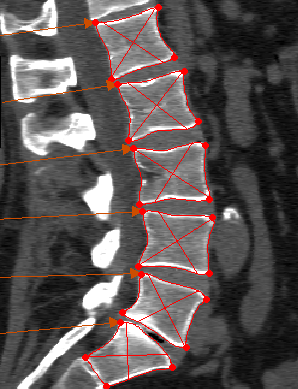 | **B**  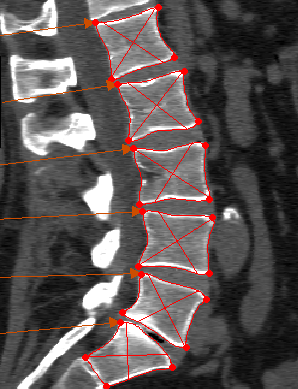 |
| --- | --- |

**Appendix 2. Study flow diagram for procedural reliability testing**

*The flow diagram illustrates the number of computed tomography images excluded from those available for reliability testing.*
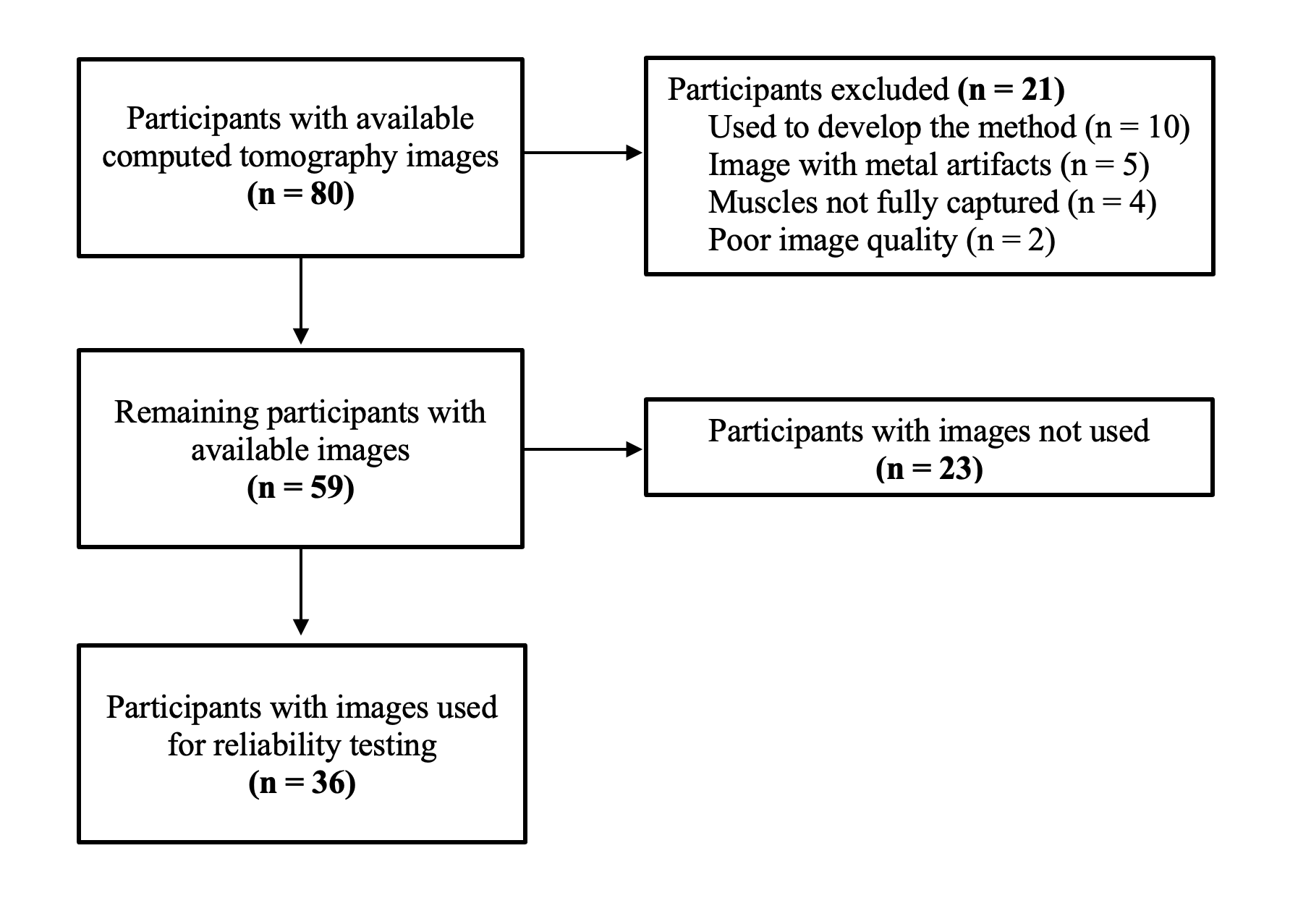

Supplement: Supplementary file 1 [file mmc1.docx]
